# Ancient grains as novel dietary carbohydrate sources in canine diets

**DOI:** 10.1093/jas/skab080

**Published:** 2021-03-25

**Authors:** Zachary T Traughber, Fei He, Jolene M Hoke, Gary M Davenport, Sandra L Rodriguez-Zas, Bruce R Southey, Maria R C de Godoy

**Affiliations:** 1 Department of Animal Sciences, University of Illinois, Urbana, IL 61801, USA; 2 Archer Daniels Midland Company, Decatur, IL 62526, USA

**Keywords:** ancient grains, digestibility, dog, microbiome, post-prandial response

## Abstract

Ancient grains are becoming an increasingly abundant carbohydrate source in the pet food market as a result of their popularity and novelty in the human market. Thus, it is imperative to evaluate the characteristics of these ingredients in vivo. Ten adult intact female beagles were used in a replicated 5 × 5 Latin square design. Five dietary treatments were evaluated containing either: rice (**CON**), amaranth (**AM**), white proso millet (**WPM**), quinoa (**QU**), or oat groats (**OG**). All diets were formulated to include 40% of the test grain and to be isonitrogenous, isocaloric, and nutritionally complete and balanced for adult dogs at maintenance. The objectives were 1) to evaluate the effects of the novel carbohydrate sources on total apparent total tract digestibility (**ATTD**), fecal microbiota, and fermentative end-product concentrations and 2) to evaluate the effects of novel carbohydrate sources on the postprandial glycemic and insulinemic responses in healthy adult dogs. All diets were well accepted by the dogs and fecal scores remained within the ideal range for all treatments. In terms of ATTD, all diets were well digested by the dogs; WPM had the highest digestibility of dry and organic matter in contrast with dogs fed the other treatments (*P* < 0.05). Additionally, ATTD of total dietary fiber was highest for WPM (72.6%) in contrast with QU (63.5%) and CON (50.8%) but did not differ from AM (65.7%) and OG (66.6%). Dogs fed AM or OG had greater (*P* < 0.05) fecal concentrations of total short-chain fatty acids, as well as propionate and butyrate concentrations, than CON. Ancient grain inclusion appears to beneficially shift fecal microbial populations, with increases in relative abundances of butyrogenic bacteria (i.e., members of the Lachnospiraceae family) observed for OG and reductions in Fusobacteriaceae for both AM and OG when compared with CON. Postprandial glycemic and insulinemic responses did not differ among treatments. Together, these data suggest that ancient grains can be included up to 40% of the diet while eliciting beneficial effects on the overall host health without detrimentally affecting nutrient digestibility.

## Introduction

Pets have become more integrated into family life than in years past, and, in doing so, owners see themselves less as pet owners and more as pet parents (Owens and Grauerholz, 2019). This change in the owner/parent mindset has led to the projection of human perceptions onto the pet food market (i.e., non-genetically modified organisms, gluten-free, and byproduct-free; Case, 2014). Ancient grains are a prime example of an increasingly popular trend in human and, subsequently, pet foods. This is due to the marketing of these ingredients as sustainable, gluten- and GMO-free, fiber-rich alternatives to common grains. They also have a positive label appeal on pet foods as they become more prevalent in commercial diets and treats despite limited scientific information on their nutritional value and functional properties for dogs and cats.

Ancient grains are “primitive grains, not subject to any modern breeding or selection, thus retaining characteristics of their wild ancestors” (Giambanelli et al., 2013). They demonstrate high resiliency, require less fertilization, and show increased adaptability (Boukid et al., 2018) in aggressive climates where most of the common crops cannot thrive. Dietary inclusion of ancient grains has potential health benefits for humans and animals alike. Sofi et al. (2013) reported that the consumption of food products containing the ancient wheat variety, Kamut, led to a reduction in metabolic risk factors in humans. They reported that participants consuming Kamut-containing products had decreased total and low-density lipoprotein cholesterol, increased serum potassium and magnesium, and an ameliorated inflammatory profile. Postprandial glucose responses and decreased appetite have also been attributed to dietary pearl millet inclusion in human foods (Alyami et al., 2019). These beneficial responses have strong implications in the current pet obesity epidemic. Recent research has demonstrated promising reductions in metabolic and cardiovascular risk factors and colon cancer cell proliferation inhibition in vitro (Vilcacundo et al., 2018). Ancient grains have also been shown to have antioxidative properties (Inglett et al., 2015) and nephroprotective properties in diabetic rats (Shobana et al., 2010). 

Research on the potential health effects of a high inclusion of the present ancient grains in pet food is limited. Thus, the objectives were 1) to evaluate the effects of the novel carbohydrate sources on total apparent total tract digestibility (**ATTD**), fecal microbiota, and fermentative end-product concentrations and 2) to evaluate the effects of novel carbohydrate sources on the postprandial glycemic and insulinemic responses in healthy adult dogs. It was hypothesized that the consumption of ancient grain-based diets would beneficially shift fermentative end products and fecal microbial populations and lower glycemic response with no detrimental effect on nutrient digestibility or the overall animal health.

## Materials and Methods

All animal care procedures were approved by the University of Illinois Institutional Animal Care and Use Committee prior to animal experimentation (Protocol # 17135). All methods were performed in accordance with the United States Public Health Service Policy on Humane Care and Use of Laboratory Animals.

### Animals

Ten adult female beagles (mean age = 4.2 + 1.14 yr; mean weight = 11.1 ± 1.17 kg; mean body condition score [**BCS**] = 5.7 ± 0.7) were used in a replicated 5 × 5 Latin square design. The dogs were housed individually in pens during diet adaptation phases and then were placed in metabolic cages during the collection phase. Dogs had nose-to-nose contact with dogs in adjacent runs and visual contact with all dogs in a temperature-controlled room at the Veterinary Medicine Basic Sciences Building. A 14:10 (L:D) h schedule was maintained throughout the study. All dogs were fed twice daily at 0800 and 1600 hours with unlimited access to water. Dogs were fed to maintain body weight with diet intake and refusals recorded for each meal. Weekly body weight and BCS assessments occurred prior to morning feeding. The BCS of each dog was assessed subjectively using a 9-point scale with “1” being severely malnourished, “5” being ideal, and “9” being severely overweight (Laflamme, 1997).

### Diets

Five dietary treatments were used in this study: rice (**CON**), amaranth (**AM**), white proso millet (**WPM**), quinoa (**QU**), and oat groats (**OG**). All diets were formulated to be isonitrogenous and isocaloric as well as to be nutritionally complete and balanced for adult dogs at maintenance (AAFCO, 2017). Diets were formulated with 40% of the test grain included at the expense of rice, poultry byproduct meal, and poultry fat (Table 1). Rice flour was utilized due to its glycemic potential as a positive control for analyzed glycemic responses in addition to its commonality as an ingredient in pet foods. Test diets were formulated to contain approximately 10% rice flour as a starch source to aid in expansion during extrusion as well as to ensure consistency between treatment groups and reduce potential confounding of data. Nutrient targets for each diet were 94% dry matter (**DM**), 32% crude protein (**CP**), 14% acid hydrolyzed fat (**AHF**), 10% total dietary fiber (**TDF**), 9% ash, and 5.0 kcal/g gross energy (**GE**). Diets were produced by Wenger Manufacturing (Wenger Manufacturing Inc., Sabetha, KS) and processing and extrusion parameters for test diets can be found within Table 2. Processing conditions were maintained to ensure consistent kibble size and density among the final products.

**Table 1.** Ingredient composition of ancient grain-based diets fed to adult dogs^1^

	Treatment^2^				
Item^3^, % as-is basis	CON	AM	WPM	QU	OG
Amaranth	—	40.0	—	—	—
White proso millet	—	—	40.0	—	—
Quinoa	—	—	—	40.0	—
Oat groats	—	—	—	—	40.1
Rice flour	40.2	10.0	9.8	8.3	10.0
Turkey byproduct meal	32.6	27.8	27.8	28.1	26.9
Poultry fat	8.0	7.6	7.7	8.0	7.0
Cellulose, Solka floc	5.1	0.1	0.6	1.8	1.0
Corn	5.0	5.0	5.0	5.0	5.0
Corn gluten meal, 60%	5.0	5.0	5.0	5.0	5.0
Dicalcium phosphate, 18.5%	1.3	1.9	0.4	0.0	2.1
Digest, dry	1.0	1.0	1.0	1.0	1.0
Calcium carbonate	—	—	1.0	1.1	—
Vitamin/mineral premix^4^	0.6	0.6	0.6	0.6	0.6
Potassium chloride	0.6	0.6	0.6	0.6	0.6
Salt	0.3	0.3	0.3	0.3	0.3
Choline chloride, 60%	0.2	0.2	0.2	0.2	0.2
Antioxidant	0.02	0.02	0.02	0.02	0.02

^1^All diets were formulated to be nutritionally complete and balanced for adult maintenance.

^2^CON, control; AM, amaranth; WPM, white proso millet; QU, quinoa; OG, oat groats.

^3^All ingredients provided by Archer Daniels Midland Company, Quincy, IL.

^4^Provided per kg diet: 10.8 mg copper (CuSO_4_), 0.36 mg selenium (Na_2_SeO_3_), 150 mg zinc (ZnSO_4,_ ZnO), 2,562.8 IU vitamin A, 254 IU vitamin D3, and 32.1 IU vitamin E.

**Table 2.** Average single screw extruder (Wenger X-115) processing conditions for dietary treatments containing selected ancient grains

	Treatment				
Measurement	CON	AM	WPM	QU	OG
Raw material					
Dry recipe density, kg/m^3^	601.0	536.0	568.0	564.0	495.0
Dry recipe rate, kg/h	495.0	502.0	487.0	490.0	482.0
Feeder speed, rpm	45.3	45.5	45.6	42.0	53.6
Preconditioner					
Mixing intensity, %	30.0	30.0	30.0	30.0	30.0
Large side speed, rpm	263.0	263.0	263.0	263.0	263.0
Small side speed, rpm	377.0	377.0	377.0	377.0	377.0
Cylinder steam, kg/h	40.1	40.1	40.1	40.0	40.0
Cylinder water, kg/h	80.3	79.9	89.6	80.3	80.2
Cylinder discharge temp, °C	83.0	81.0	82.0	80.0	78.0
Extruder					
Speed, rpm	370.0	430.0	475.0	500.0	520.0
Motor load, %	56.4	50.8	45.4	64.7	50.8
Motor power, kW	24.4	24.5	22.3	36.6	27.9
Knife speed, rpm	1,201.0	1,200.0	1,500.0	1,501.0	1,501.0
Zone 1 temp, °C	90.0	87.0	87.0	92.0	91.0
Zone 2 temp, °C	95.0	95.0	95.0	96.0	95.0
Zone 3 temp, °C	100.0	100.0	99.0	111.0	101.0
Zone 4 temp, °C	108.0	106.0	108.0	117.0	103.0
Zone 5 temp, °C	112.0	110.0	110.0	108.0	111.0
Conehead pressure, KPA	209.0	219.0	246.0	250.0	328.0
Specific mechanical energy	49.2	48.8	45.9	74.6	58.0
Dryer					
Zone 1 temp, °C	133.0	129.0	135.0	135.0	134.0
Zone 2 temp, °C	68.0	66.0	72.0	71.0	72.0
Zone 3 temp, °C	99.0	91.0	98.0	94.0	98.0
Retention time—pass 1, min	21.0	20.0	20.0	20.0	20.0
Retention time—pass 2, min	9.0	8.0	6.0	6.0	8.0
Exhaust 1 temp, °C	81.0	74.0	81.0	79.0	81.0
Final product					
Extruder discharge density	396.0	404.0	396.0	396.0	400.0

### Experimental design and sample collection

Dogs were randomly assigned to one of the five experimental treatments. Dogs were subsequently randomized into one of the two groups, offset by 1 d, to facilitate the postprandial glycemic response measurements. The study consisted of five periods with each period comprised of a 10-d diet adaptation phase, followed by 4 d of fecal and urine collections, and 1 d to conduct the postprandial glycemic response.

On the last day of each period, when the postprandial glycemic response test was conducted, a 6-mL fasted (12 h) blood sample was collected via jugular venipuncture and was used as a baseline indicator for a complete blood count, serum chemistry, glucose, and insulin. Serum chemistry and complete blood count were used to assess the health of the dogs throughout the study and were analyzed by the University of Illinois Veterinary School Diagnostics Laboratory using a Hitachi 911 clinical chemistry analyzer (Roche Diagnostics, Indianapolis, IN). All dogs were then allowed 15 min to consume their total daily food allowance. Dogs that failed to complete their full daily allowance in the allotted time were removed from the postprandial glycemic assessment for that day. Upon the immediate completion of each individual feeding, a 4-mL blood sample was collected every 30 min for the first 240 min (4 h) with a final sample collected at the 360 min (6 h) time point. Time point samples were evaluated for whole-blood glucose and serum insulin concentrations. A period of 6 h was chosen to evaluate postprandial glycemic response, as it would allow sufficient time for the dogs to digest their food and enter the absorptive stage.

Total fecal samples were collected over a 4-d period following a 10-d diet adaptation phase and ended on the morning of the glycemic response analysis. Feces were scored on a 5-point scale (1 = hard, dry pellets, small hard mass; 2 = hard formed, remains firm and soft; 3 = soft, formed and moist stool, retains shape; 4 = soft, unformed stool, assumes shape of container; and 5 = watery, liquid that can be poured). Fresh fecal samples were collected within 15 min of defecation and were subjectively scored, analyzed for pH, and subsampled for subsequent DM, short-chain fatty acids (**SCFA**), branched-chain fatty acids (**BCFA**), phenols, indoles, ammonia concentrations, and fecal microbiota analyses. All fecal subsamples were immediately frozen at −20 °C. A separate sample was also collected and stored at −80 °C for the determination of microbial populations. Total urine was collected in containers containing 10 mL 2 N hydrochloric acid for immediate acidification upon urination. Acidified urine samples were subsampled, pooled, and stored at −20 °C until analysis.

Blood was collected via a cephalic catheter (Exelint International Co., Redondo Beach, CA) and placed into collection tubes for serum (BD Vacutainer, SST, Franklin Lakes, NJ) and plasma (BD Vacutainer, K_2_ EDTA 3.6 mg, Franklin Lakes, NJ). Samples were centrifuged (1,300 × *g* at 4 °C) and supernatants were pipetted into cryovials and immediately frozen at −80 °C and stored until further analysis.

### Sample preparation and chemical analysis

Diets were analyzed during processing for percent starch gelatinization, total starch, and gelatinized starch by Wenger Manufacturing (Wenger Manufacturing Inc., Sabetha, KS). Food and fecal samples were used to determine the ATTD of macronutrients. Fecal samples were dried at 55 °C in a forced-air oven and ground in a Wiley mill (model 4; Thomas Scientific, Swedesboro, NJ) through a 2-mm screen. Diet and fecal samples were analyzed for DM and ash according to AOAC (2006; methods 934.01 and 942.05) with organic matter (**OM**) calculated by difference. CP content of the diets and fecal samples was calculated from Leco (TruMac N, Leco Corporation, St. Joseph, MI) total nitrogen values according to AOAC (2006; method 992.15). Total lipid content was determined by acid hydrolysis followed by ether extraction according to the methods of the American Association of Cereal Chemists (1983) and Budde (1952). Diet and fecal TDF contents were analyzed according to Prosky et al. (1992). Diet, fecal, and urine samples were analyzed for GE by bomb calorimeter (Model 6200, Parr Instruments Co., Moline, IL). Urine GE values were used to calculate metabolizable energy (**ME**).

Gas chromatography was used to analyze fecal samples for SCFA and BCFA according to the methods of Erwin et al. (1961) and Goodall and Byers (1978). Phenol and indole concentrations were measured according to the method of Flickinger et al. (2003). Fecal ammonia concentrations were determined using the method of Chaney and Marbach (1962).

### DNA extraction, amplification, sequencing, and bioinformatics

Total DNA was extracted from fresh fecal samples using Mo-Bio PowerSoil kits (MO BIO Laboratories, Inc., Carlsbad, CA) and DNA concentration was quantified using a Qubit 2.0 Fluorometer (Life Technologies, Grand Island, NY). Amplification of the 16S rRNA gene was completed using a Fluidigm Access Array (Fluidigm Corporation, South San Francisco, CA) in combination with Roche High Fidelity Fast Start Kit (Roche, Indianapolis, IN). The primers 515F (5′-GTGCCAGCMGCCGCGGTAA-3′) and 806R (5′-GGACTACHVGGGTWTCTAAT-3′) that target a 291 bp-fragment of V4 region were used for amplification (primers synthesized by IDT Corp., Coralville, IA; Caporaso et al., 2012). Fluidigm-specific primer forward (CS1) and reverse (CS2) tags were added according to the Fluidigm protocol. Fragment Analyzer (Advanced Analytics, Ames, IA) was used to confirm the quality of amplicons’ regions and sizes. A DNA pool was generated by combining equimolar amounts of the amplicons from each sample. The pooled samples were then size selected on a 2% agarose E-gel (Life Technologies, Grand Island, NY) and extracted using Qiagen gel purification kit (Qiagen, Valencia, CA). Cleaned size-selected pooled products were run on an Agilent Bioanalyzer to confirm appropriate profile and average size. Illumina sequencing was performed on a MiSeq using v3 reagents (Illumina Inc., San Diego, CA) at the W. M. Keck Center for Biotechnology at the University of Illinois. Fluidigm tags were removed using FASTX-Toolkit (version 0.0.14), and sequences were analyzed using QIIME 2.0 (Caporaso et al., 2010) and DADA2 (version 1.14; Callahan et al., 2016). High-quality (quality value ≥ 20) sequence data derived from the sequencing process were demultiplexed. Sequences were then clustered into operational taxonomic units (**OTU**s) using opened-reference OTU picking against the SILVA 138 reference OTU database with a 97% similarity threshold (Quast et al., 2013). Singletons (OTUs observed fewer than 2 times) and OTUs having less than 0.01% of the total observations were discarded. A total of 2,919,615 reads were obtained, with an average of 58,392 reads per sample (range = 36,137 to 87,436). The dataset was rarified to 36,130 reads for the analysis of diversity and species richness. Principal coordinates analysis were performed, using both weighted and unweighted unique fraction metric (**UniFrac**) distances that measured the phylogenetic distance between sets of taxa in a phylogenetic tree as the fraction of the branch length of the tree, on the 97% OTU composition and abundance matrix (Lozupone and Knight, 2005).

### Postprandial glucose and insulin measurements

Glucose was analyzed immediately after the collection via an AlphaTRAK 2 canine and feline glucose meter (Zoetis, Parsippany, NJ). A single drop of blood was introduced to an AlphaTRAK 2 test strip (Zoetis, Parsippany, NJ), and blood glucose was recorded. Mercodia Canine Insulin enzyme-linked immunosorbent assay (Mercodia AB, Uppsala, Sweden) was used to analyze serum supernatant for insulin concentration at each time point. Both the positive area under the curve (**AUC**) and the positive incremental AUC (**IAUC**), evaluated as a change from baseline, for glucose and insulin values were calculated using GraphPad Prism 5 Software (GraphPad Software, Inc., San Diego, CA). The relative glucose response (**RGR**) and relative insulinemic response (**RIR**) of the test diets were calculated as (IAUC_test_) / (IAUC_control_) × 100% with the control diet assigned a reference value of 1.00.

### Statistical analysis

All data were analyzed using SAS (SAS Institute INC., version 9.4, Cary, NC); ATTD and fecal metabolite data were analyzed with PROC MIXED, while glycemic and insulinemic response data were analyzed using PROC GLIMMIX. Diet was a fixed effect and dog was a random effect for all analyses. The normality of residuals was verified using PROC UNIVARIATE. Differences among treatments were determined using a Fisher-protected least significant difference test with a Tukey adjustment to control for type-1 experiment-wise error. Data are presented as LSMeans with statistical significance set at *P* < 0.05 and trends defined as 0.05 < *P* < 0.10.

## Results

### Diets, food intake, and fecal characteristics

Moisture content ranged from 4.3% to 7.7%, CP varied between 31.4% and 33.1%, AHF was between 12.3% and 14%, TDF ranged from 10.7% to 13.4%, the average ash value was 9.5%, and GE averaged 5.0 kcal/g on a dry matter basis (**DMB**) for all diets (Table 3).

**Table 3.** Chemical composition and starch gelatinization for ancient grain-based diets fed to adult dogs

	Treatment^1^				
Item, %	CON	AM	WPM	QU	OG
Moisture^2^	6.5	6.4	7.2	5.3	7.7
Moisture^3^	5.4	5.7	7.7	4.3	4.4
Gelatinization^2^	88.5	85.5	88.0	96.6	91.6
	**-----** %, DMB -----				
Total starch^2^	35.9	35.9	39.5	36.2	36.1
Gelatinized starch^2^	31.8	30.7	34.7	35.0	33.0
CP^3^	31.3	32.6	32.4	31.4	33.1
AHF^3^	14.4	14.5	12.3	14.8	14.6
TDF^3^	12.4	11.9	10.7	12.9	13.4
Ash^3^	9.3	9.5	9.5	9.4	9.3
GE^2^, kcal/g	5.0	5.0	4.9	5.1	5.1

^1^CON, control; AM, amaranth; WPM, white proso millet; QU, quinoa; OG, oat groats..

^2^Analyzed on the day of extrusion.

^3^Analyzed at the onset of feeding trial.

Starch gelatinization varied among diets ranging from 85.5% for AM to 96.6% for QU (Table 3). The total starch content of the diets ranged from 39.5% for WPM to 35.9% for both CON and AM. Gelatinized starch also ranged from 31% to 35%.

Food intake did not differ among treatments. Fecal scores were higher (*P* < 0.05) for QU (3.0) than for dogs fed CON, with all other treatments showing no differences (avg. 2.8; Table 4). Fecal output on an as-is basis was greater (*P* < 0.05) for dogs fed QU and AM and lowest for dogs fed WPM. When expressed on a DMB, fecal output by dogs fed OG, QU, and AM were all significantly higher *(P* < 0.05) than WPM.

**Table 4.** Food intake and fecal characteristics of adult dogs fed diets containing selected fiber/carbohydrate sources

	Treatments^1^					
Item	CON	AM	WPM	QU	OG	SEM
Food intake						
g/d, DMB	155.5	154.7	153.6	160.4	158.7	3.45
Fecal output						
Fecal pH	7.0	6.7	7.0	7.0	6.7	0.15
Fecal score^2^	2.7^b^	2.9^ab^	2.9^ab^	3.0^a^	2.9^ab^	0.10
Fecal output, as-is (g/d)	51.2^bc^	67.8^a^	45.7^c^	68.8^a^	62.1^ab^	4.70
Fecal output, DMB (g/d)	26.0^ab^	26.5^a^	21.1^b^	29.0^a^	27.2^a^	1.85

^1^CON, control; AM, amaranth; WPM, white proso millet; QU, quinoa; OG, oat groats.

^2^Fecal score: 2 = hard formed, remains firm and soft; 3 = soft, formed and moist stool, retains shape.

^a–c^Means in the same row without common superscript letters are different (*P* < 0.05).

### Determination of ATTD and energy content of diets

**Figure 1.**
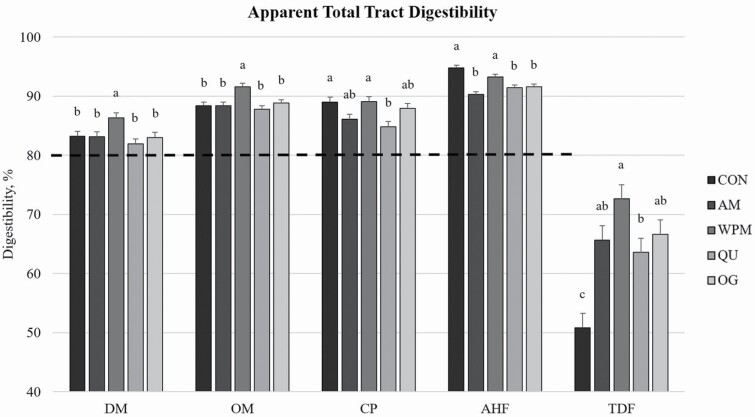
Macronutrient ATTD of diets comprised primarily of selected fiber/carbohydrate sources fed to adult dogs. All diets were highly digestible (>80%). ^a,b^Bars in the same group without common letters are different (*P* < 0.05).

All diets had TTD values greater than 80% (Figure 1) for all macronutrient categories other than TDF. Treatment group WPM had significantly higher DM, OM, and TDF digestibility (86.3%, 91.6%, and 72.6%, respectively) than all other treatment groups. Both WPM and CON had significantly higher digestibilities of CP (89.1% and 89.0%, respectively) and AHF (93.2% and 94.8%, respectively) than all other treatment groups. CON had the lowest (*P* < 0.05) TDF digestibilities among all treatment groups. Excluding TDF, the ATTD of all analyzed macronutrients for dogs fed QU was numerically lowest among treatments (81.9%, 87.8%, 84.8%, and 91.4% for DM, OM, CP, and AHF, respectively).

**Figure 2.**
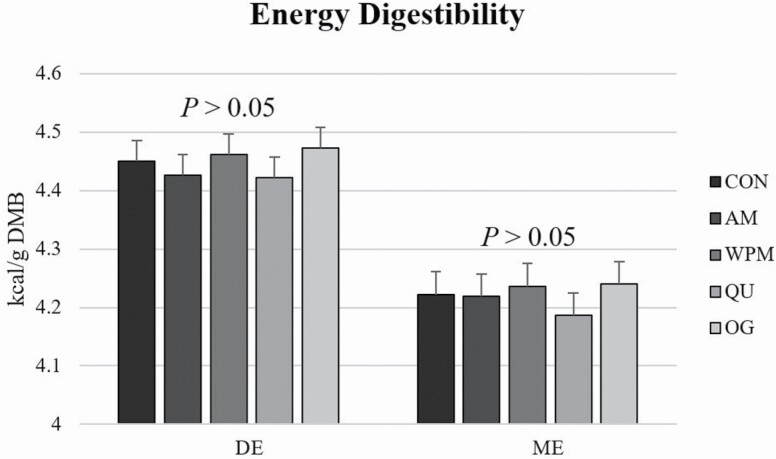
Energy digestibility of diets comprised primarily of selected fiber/carbohydrate sources fed to adult dogs.

No differences (*P* > 0.05) were observed in digestible energy (**DE**) or ME contents among treatment groups (Figure 2), with all diets having approximately 4.5 kcal/g DE and 4.2 kcal/g ME (DMB).

### Fecal metabolite concentrations

Dogs fed AM and OG had significantly greater fecal concentrations of total SCFA (406.6 and 363.0 umol/g DMB, respectively) as well as concentrations of propionate (124.0 and 111.3 umol/g DMB, respectively) and butyrate (64.1 and 48.5 umol/g DMB, respectively) than dogs fed CON (222.5, 59.4, and 29.5 umol/g DMB, respectively). Fecal acetate concentrations were greater (*P* < 0.05) for dogs fed AM than CON dogs (218 and 133.3 umol/g DMB, respectively; Table 5). Fecal concentrations of total BCFA and isobutyrate were greater (*P* < 0.05) for dogs fed OG (22.1 and 8.8 umol/g DMB, respectively) than for dogs fed AM (16.9 and 6.3 umol/g DMB, respectively) and QU (16.0 and 6.5 umol/g DMB, respectively) with fecal isovalerate concentrations of dogs fed OG (12.6 umol/g DMB) being greater (*P* < 0.05) than QU (9.0 umol/g DMB; Table 5). Fecal valerate concentrations for dogs fed AM and OG were greater (*P* < 0.05) than for dogs fed CON. Fecal concentrations of phenols were lower (*P* < 0.05) for dogs fed CON than dogs fed any of the ancient grain-containing diets, with no differences noted among treatments for indoles (Table 5). When total phenols and indoles were combined, only values for dogs fed AM, WPM, and QU were lower (*P* < 0.05) than those for dogs fed CON. Fecal ammonia concentrations were significantly greater (*P* < 0.05) for dogs fed OG than for those fed WPM and QU (Table 5).

**Table 5.** Fecal fermentative end-product concentrations for adult dogs fed diets containing selected fiber/carbohydrate sources^1^

Item, DMB	CON	AM	WPM	QU	OG	SEM
Total SCFA, umol/g	222.5^b^	406.6^a^	315.4^ab^	310.5^ab^	363.0^a^	25.38
Acetate	133.6^b^	218.5^a^	191.8^ab^	199.5^ab^	203.2^ab^	18.59
Propionate	59.4^c^	124.0^a^	82.2^bc^	79.7^bc^	111.3^ab^	10.45
Butyrate	29.5^c^	64.1^a^	41.4^bc^	31.2^bc^	48.5^ab^	4.66
Total BCFA, umol/g	19.3^ab^	16.9^b^	19.0^ab^	16.0^b^	22.1^a^	1.76
Isobutyrate	7.7^ab^	6.3^b^	7.3^ab^	6.5^b^	8.8^a^	0.73
Isovalerate	11.3^ab^	9.7^ab^	11.2^ab^	9.0^b^	12.6^a^	1.03
Valerate	0.3^c^	0.9^a^	0.5^bc^	0.5^bc^	0.7^ab^	0.10
Ammonia, mg/g	2.4^ab^	2.4^ab^	2.2^b^	2.0^b^	2.8^a^	0.20
Total PI^2^, ug/g	358.3^a^	248.4^b^	250.6^b^	233.2^b^	300.8^ab^	36.18
Phenols	96.8^a^	27.1^b^	28.5^b^	15.3^b^	29.6^b^	19.17
Indoles	261.5	221.3	222.1	217.9	271.2	23.57

^1^CON, control; AM, amaranth; WPM, white proso millet; QU, quinoa; OG, oat groats.

^2^Total PI, total phenols and indoles.

^a–c^Means in the same row without common superscript letters are different (*P* < 0.05).

### Fecal microbial populations

**Figure 3.**
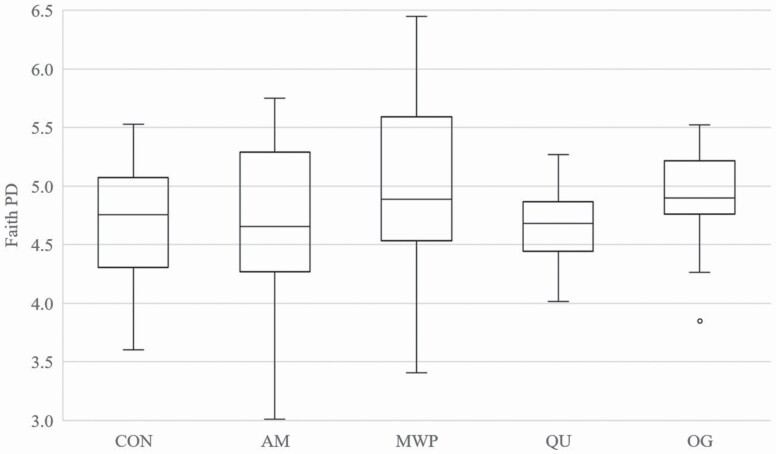
Fecal microbial alpha-diversity analysis of dogs fed diets comprised primarily of selected fiber/carbohydrate sources. Within-sample diversity measured by Faith’s phylogenetic diversity (**PD**) suggested that species evenness within a sample was not affected by treatment (*P* < 0.05).

**Figure 4.**
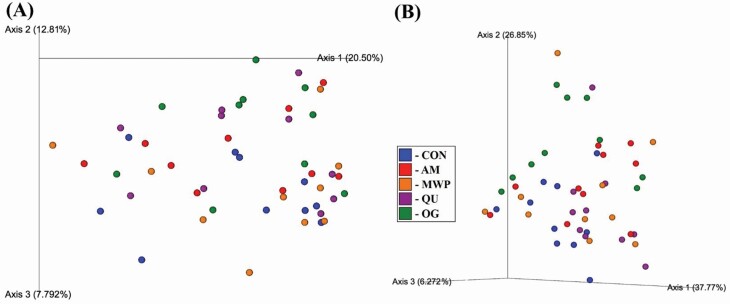
Fecal microbial alpha-diversity analysis of dogs fed diets comprised primarily of selected fiber/carbohydrate sources. Principal coordinate analysis (PCoA) plots of (A) unweighted and (B) weighted UniFrac distances of fecal microbial communities were not altered by treatment (*P* < 0.05).

**Figure 5.**
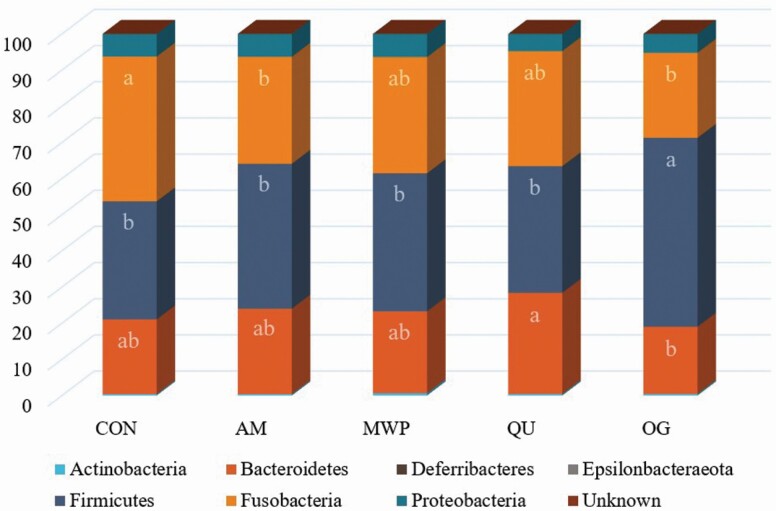
Fecal microbial phyla (%, total sequences) of dogs fed diets comprised primarily of selected fiber/carbohydrate sources. ^a,b^Bars within the same phylum without common letters are different (*P* < 0.05).

Alpha diversity (Figure 3) and beta diversity (Figure 4) did not differ among treatments. However, the relative abundance of fecal bacterial phyla and families with significant differences among dietary treatments is displayed in Table 6, and the relative abundance of significant genera is displayed in Table 7. Seven total bacterial phyla were observed with Bacteroidetes, Firmicutes, and Fusobacteria as the three most predominant phyla, collectively accounting for ~90% of all sequences (Figure 5). Dogs consuming OG had lower (*P* < 0.05) populations of Bacteroidetes than those consuming QU (18.6% and 28.0%, respectively) in addition to the greatest (*P* < 0.05) concentrations of Firmicutes (52.2%) among all treatments. Dogs consuming CON had greater (*P* < 0.05) populations of Fusobacteria (40.0%) than those consuming AM or OG (29.6% and 23.5%, respectively).

**Table 6.** Relative abundance of fecal bacterial phyla and families of adult dogs fed diets containing selected carbohydrate sources

		Treatment^1^					
Phyla, % sequences	Family	CON	AM	WPM	QU	OG	SEM
Bacteroidetes		20.66^ab^	23.58^ab^	22.62^ab^	27.96^a^	18.58^b^	2.519
	Bacteroidaceae	14.79^bc^	16.12^ab^	13.23^bc^	20.01^a^	10.83^c^	1.39
	Tannerellaceae	0.78^b^	0.74^b^	1.42^ab^	1.57^a^	0.83^b^	0.302
Firmicutes		32.64^b^	40.03^b^	38.16^b^	34.99^b^	52.21^a^	3.413
	Lactobacillaceae	0.32^b^	0.98^ab^	0.55^ab^	0.10^b^	2.48^a^	0.672
	Streptococcaceae	0.23^ab^	0.93^ab^	0.29^ab^	0.11^b^	1.48^a^	0.346
	Lachnospiraceae	15.26^y^	16.65^xy^	17.17^xy^	17.62^y^	22.76^x^	1.873
	Erysipelotrichaceae	4.41^b^	5.25^b^	5.71^ab^	3.95^b^	8.07^a^	0.728
	Veillonellaceae	0.58^bc^	2.39^a^	0.55^bc^	0.13^c^	1.68^ab^	0.393
Fusobacteria		40.03^a^	29.57^b^	32.13^ab^	31.83^ab^	23.52^b^	2.847
	Fusobacteriaceae	40.03^a^	29.57^b^	32.13^ab^	31.83^ab^	23.52^b^	2.847

^1^CON, control; AM, amaranth; WPM, white proso millet.

^a–c^Means in the same row without common superscript letters are different (*P* < 0.05).

^x,y^Means in the same row without common superscript letters are different (*P* < 0.10).

**Table 7.** Relative abundance of fecal bacterial genera of adult dogs fed diets containing selected carbohydrate sources

		Treatment^1^					
Phyla, % sequences	Genus	CON	AM	WPM	QU	OG	SEM
Bacteroidetes	*Bacteroides*	14.79^bc^	16.12^ab^	13.23^bc^	20.01^a^	10.83^c^	1.390
	*Prevotella 9*	0.53^b^	2.52^a^	2.06^ab^	1.79^ab^	2.46^a^	0.691
	*Parabacteroides*	0.78^ab^	0.74^b^	1.42^ab^	1.57^a^	0.83^ab^	0.302
Firmicutes	*Lactobacillus*	0.32^b^	0.98^ab^	0.55^ab^	0.10^b^	2.48^a^	0.672
	*Streptococcus*	0.23^b^	0.93^ab^	0.29^ab^	0.11^b^	1.48^a^	0.346
	*Lachnoclostridium*	1.34^a^	0.11^c^	0.93^ab^	0.41^bc^	0.27^bc^	0.223
	*Lachnospira*	0.07^b^	0.05^b^	0.00^b^	1.65^a^	0.00^b^	0.124
	Lachnospiraceae*, undefined*	7.28^b^	8.58^b^	9.02^b^	9.43^ab^	13.51^a^	1.226
	*Romboutsia*	0.93^bc^	1.74^ab^	1.31^abc^	0.88^c^	1.97^a^	0.256
	*Erysipelotrichaceae UCG-003*	0.15^b^	0.60^ab^	0.27^b^	0.00^b^	1.90^a^	0.384
Firmicutes	*Holdemanella*	0.15^b^	0.70^ab^	0.63^ab^	0.96^a^	0.66^ab^	0.219
	*Turicibacter*	0.14^b^	0.80^a^	0.52^ab^	0.14^b^	0.52^ab^	0.132
	*Megamonas*	0.58^bc^	2.32^a^	0.55^bc^	0.13^c^	1.68^ab^	0.381
Fusobacteria	*Fusobacterium*	40.03^a^	29.57^b^	32.13^ab^	31.83^ab^	23.52^b^	2.847

^1^CON, control; AM, amaranth; WPM, white proso millet.

^a–c^Means in the same row without common superscript letters are different (*P* < 0.05).

Twenty-four total bacterial families were observed with the predominant fecal families, Bacteroidaceae (Bacteroidetes/Chlorobi group), Lachnospiraceae (Firmicutes), and Fusobacteriaceae (Fusobacteria), accounting for approximately 65% of all sequences. Bacteroidaceae relative abundance was greatest (*P* < 0.05) for dogs consuming QU (20.0%) and among the lowest for those fed OG (10.8%). Lactobacillaceae, Streptococcaceae, and Erysipelotrichaceae were among the greatest (*P* < 0.05) for dogs fed OG (2.5%, 1.5%, and 8.1%, respectively) compared with all other treatments. Dogs consuming CON had a greater (*P* < 0.05) relative abundance of Fusobacteriaceae (40.0%) compared with AM and OG (range: 29.6 and 23.5, respectively).

Fifty-two total bacterial genera were observed with *Fusobacterium* (Firmicutes), *Bacteroides* (Bacteroidetes), and undefined Lachnospiraceae (Firmicutes), accounting for approximately 55% of all sequences. Relative abundance of *Bacteroides* was lower (*P* < 0.05) in dogs fed OG (10.8%) than those fed QU or AM (20.0% and 16.2%, respectively). A greater (*P* < 0.05) relative abundance of *Prevotella 9* was observed in feces of dogs fed AM and OG (2.5% and 2.5%, respectively) compared with dogs fed CON (0.5%). Greater (*P* < 0.05) relative abundance of *Lactobacillus* was observed in feces of dogs fed OG (2.5%) than dogs fed CON or QU (0.3% and 0.1%, respectively). A greater relative abundance of Undefined Lachnospiraceae was observed in dogs fed OG (13.5%) compared with CON, AM, and WPM (7.7%, 8.6%, and 9.0%, respectively).

### Glycemic and insulinemic responses

No significant differences (*P* > 0.05) were observed among treatment groups for any parameters: IAUC, RGR, time to peak, peak value, or peak area (Table 8). When evaluating the insulinemic response, this same pattern was present for dogs fed OG for all parameters except RIR (50,144 min * mmol/L, 135.3 min, 334.06 mmol/L, and 50,24 min * mmol/L for IAUC, time to peak, peak value, and peak area, respectively; Table 8). For RIR, values for dogs fed WPM tended to be greater (*P* < 0.10) than CON (1.83 and 1.00, respectively) with no differences observed among any other treatment groups.

**Table 8.** Serum glucose and insulin responses for adult dogs fed diets containing selected fiber/carbohydrate sources^1^

Item^2^	CON	AM	WPM	QU	OG	SEM
Glucose, mmol/L						
IAUC, min * mmol/L	207.1	268.1	219.2	282.5	151.9	43.24
RGR^3^	1.0	1.4	1.3	1.6	0.9	0.25
Time to peak, min	129.8	144.0	130.0	156.7	127.2	25.91
Peak value, mmol/L	1.19	1.46	1.59	1.61	1.12	0.196
Peak area, min * mmol/L	201.8	258.3	197.5	268.7	134.8	44.62
Insulin, pmol/L						
IAUC, min * pmol/L	53,115	62,448	82,650	69,205	50,144	11,800.4
RIR^3^	1.0^y^	1.4^xy^	1.8^x^	1.6^xy^	1.2^xy^	0.32
Time to peak, min	179.5	162.0	154.9	178.8	135.3	28.57
Peak value, pmol/L	392.8	380.9	468.2	459.3	334.1	58.87
Peak area, min * p mol/L	52,312	61,199	78,428	73,909	50,249	11,195.3
Insulin:glucose						
AUC	14,413^xy^	16,585^xy^	18,285^x^	16,554^xy^	13,970^y^	1,889.3
Relative ratio^4^	1.00	1.19	1.28	1.16	1.01	0.100
IAUC	347.2	327.8	524.7	409.1	629.4	181.13
Relative ratio^3^	1.00	1.34	0.90	0.87	1.69	0.335
Baseline values						
Glucose, mmol/L	90.2	90.0	92.6	89.5	92.9	2.73
Insulin, pmol/L	83.5	93.2	67.8	68.4	77.5	15.00
Ins:Glu	16.6	18.5	13.2	13.6	14.8	2.83

^1^CON, control; AM, amaranth; WPM, white proso millet; QU, quinoa; OG, oat groats.

^2^Only the positive IAUC was used; any area under baseline was ignored.

^3^ $(IAUC_{test})/(IAUC_{control})\times 100\%.$

^4^ $\left(AUC_{test}\right)/\left(AUC_{control}\right)\times 100\%.$

^x,y^Means in the same row without common letters are different (*P* < 0.10).

No significant differences (*P* > 0.05) were observed among treatment groups when evaluating the ratio of insulin to glucose changes from baseline on IAUC or the relative ratio (Table 8). Removing the normalization for baseline correction, we observed a trend (*P* = 0.06) for dogs fed OG to have a lower AUC than those fed WPM, with no differences among other treatment groups (Table 8). Baseline glucose, insulin, and insulin:glucose values were not significantly different (*P* > 0.05) between treatment groups, and covariate analysis was not required (Table 8).

## Discussion

Ancient grains are becoming an increasingly popular carbohydrate source in the pet food market. These fibrous and less rapidly digestible grains may likely have potential benefits on nutrient digestibility and postprandial blood metabolite responses. The authors are unaware of any research evaluating the inclusion of these selected ancient grains as a primary ingredient in extruded canine diets. Overall, the present data suggest that the inclusion of ancient grains at 40% of a diet may elicit beneficial effects on the overall host and gut microbiome health with no detrimental effects on nutrient digestibility.

### Food intake, ATTD of macronutrients, and fecal characteristics

All diets were well accepted by all dogs and feed refusals were minimal. Food intake did not differ among treatments. This may have been due to the feeding of dogs to maintain body weight and the similar caloric content of all diets. Ancient grain inclusion did not affect starch gelatinization with the exception of a higher percentage cook for QU. While extrusion parameters were kept as constant as possible to ensure consistent physicochemical and organoleptic properties among diets, a few extruder parameters had to be adjusted in order to achieve this goal. This is because the extrusion of complex dietary matrices can affect several processing conditions. High screw speed can increase shear and result in decreased retention time—this is because starches and proteins can exert shear-thinning properties, lowering viscosity and making the dough more fluid, which may also lower specific mechanical energy. In addition, dietary fiber is likely to affect kibble expansion, thus diets with a higher content of dietary fiber may require greater shear (i.e., screw speed) to achieve similar expansion properties of diets with lower dietary fiber content. Differences in dough fluidity and retention time can also affect knife speed in order to maintain comparable kibble size. Generally, greater backpressure translates to greater motor load and barrel fill. However, this will vary based on the characteristics of the recipes being processed. In this study, barrel fill was not determined. However, it is possible that the OG had a lower melt transition temperature, which would result in lower SME compared with the QU diet due to a slightly higher protein concentration. In general, dietary proteins have a lower melt transition value compared with starches. This is certainly an area that requires further investigation, and future studies should determine melt transition temperatures to better understand the effects of heat and shear during the extrusion process of a complete and balanced diet for pet animals.

Minimal differences were observed in fecal scores among treatment groups; however, all values remained within the ideal range of 2.5 to 3.0. The large differences observed between fecal output expressed on an as-is basis compared with a DMB for dogs fed AM and QU suggest a higher water-holding capacity for these ingredients. Water-holding capacities of 147% and 131% have been previously reported for QU (Ogungbenle, 2003) and AM (Zapotoczny et al., 2006), respectively.

All diets were highly digestible despite their higher TDF content as extruded diets (>80%). ATTD values for WPM were consistently highest for all analyzed macronutrients, with ATTD for CON also among the highest for CP and AHF. The higher DM and OM digestibility of WPM may have been a result of the slightly lower TDF content accompanied by a higher starch content compared with other treatments. Values of TDF digestibility were lowest for CON compared with other treatments with QU having a lower TDF digestibility value than WPM. All diets contained similar TDF contents; thus, the reduced TDF digestibility of CON compared with other treatments may be due to the ~5% inclusion of cellulose, a highly nondigestible, non-fermentable fiber source. Twomey et al. (2002) reported CP ATTD values for rice, corn, and sorghum of 87%, 83%, and 85%, respectively, when included in extruded canine diets at similar levels as the cereal grains in the present study.

The higher fecal SCFA concentrations for dogs fed AM and OG imply an increase in fermentable dietary carbohydrate substrates escaping intestinal hydrolytic digestion, thus becoming more available for microbial hind-gut fermentation. Greater SCFA concentration values for oats compared with rice are also consistent with previous in vitro reports using canine fecal inoculum (Bednar et al., 2001). The concentrations of fecal BCFA for dogs fed OG also were consistently highest for all treatments. The authors are unaware of previous reports on high dietary oat inclusion on fecal BCFA concentration in canines.

The differences observed in total phenols and indoles concentrations are driven by differences in phenol concentration as dogs fed diets containing ancient grains had significantly lower concentrations than dogs fed the CON diet. Reductions in fecal phenol concentrations have been reported when dogs are fed increasing levels of resistant starch (Birkett et al., 1996; Heijnen et al., 1997; Muir, 1999; Muir et al., 2004). Phenols and indoles are generally regarded as non-beneficial end products of microbial fermentation (McDonald et al., 2001; Jaglin et al., 2018) despite studies showing potential indole benefits on intestinal morphology and gut health (Bansal et al., 2010; Shimada et al., 2013; Zelante et al., 2013).

Soluble and insoluble fiber fractions of the test diets were not analyzed for this study. However, previous analyses of these ingredients within the author’s lab demonstrate that OG have the highest soluble fiber content of selected ancient grains. The inclusion of soluble fiber in the diet may also contribute to a reduction in the small intestinal digestibility (Fahey et al., 1990) and increase in the gut transit time, specifically in the large intestine (Meier et al., 1993). This would provide more substrate for hind-gut bacterial fermentation, which may explain the higher concentrations of fecal metabolites for dogs fed OG.

### Fecal microbial populations

The gastrointestinal tract is populated with a diverse collection of microorganisms and complex interactions between digesta, bacteria, and host cells. The gut microbiome plays an important role to ensure host health. For example, microbial gut dysbiosis has been linked with obesity (Kieler et al., 2017), diabetes (Jergens et al., 2019), and inflammatory bowel diseases (Vázquez-Baeza et al., 2016) in dogs. It can be implied from these data that ancient grains can have small effects on fecal microbiota, which are in agreement with other canine studies investigating cereal grain products (Eisenhauer et al., 2019; Jackson and Jewell, 2019). However, species richness and diversity were not affected by treatment in this study. The relative abundance of the three predominant bacterial phyla within the present study, Bacteroidetes, Firmicutes, and Fusobacteria, is aligned with previously reported ranges (Bacteroidetes: 12% to 34%, Firmicutes: 14% to 48%, and Fusobacteria: 23% to 40%; Suchodolski et al., 2008; Middelbos et al., 2010). Thus, the 40% inclusion of ancient grains in the present study did not affect the proportions of expected bacterial phyla in fecal samples from healthy adult dogs.

Bacteroidaceae, Lachnospiraceae, and Fusobacteriaceae have been individually reported as predominant bacterial families in fecal samples of healthy dogs (Suchodolski et al., 2009; Handl et al., 2011; Bermingham et al., 2017). However, the predominance of the combination of these three families has not been previously reported. Increases in the relative abundance of Erysipelotrichaceae and *Lactobacillus* associated with whole-grain consumption support the findings of Cooper et al. (2017). An increase in the relative abundance of *Lactobacillus* was observed in the present study for dogs fed OG. This saccharolytic genus provides beneficial effects due to its inhibitory effects on the growth of *Clostridium difficile* strains (Rolfe et al., 1981; Naaber et al., 2004). Furthermore, members of the Lachnospiraceae family include many butyrogenic bacteria, which were increased with OG consumption. This may explain the higher fecal butyrate concentrations for dogs fed OG compared with CON.

*Fusobacterium* and *Bacteroides* have also been reported as predominant fecal bacterial genera in healthy dogs consuming extruded diets (Bermingham et al., 2017). The predominance of the undefined members of the Lachnospiraceae genus in fecal microbial populations has not been previously reported. An overrepresentation of *Fusobacterium* has been associated with colorectal cancers in humans (Marchesi et al., 2011; Castellarin et al., 2012; Kostic et al., 2012) while being conversely associated with healthy dogs (Vázquez-Baeza et al., 2016). Within the present study, the relative abundance of *Fusobacterium* was reduced with the consumption of AM and OG; however, they still remained the most predominant genus among all treatment groups accounting for 100% of the total reads within the Fusobacteria phylum. The increased relative abundance of *Prevotella* and *Bacteroides* has been reported in humans consuming plant-based diets (De Filippo, 2010; David et al., 2014). The increase in *Prevotella* for AM and OG and the increase in *Bacteroides* for QU compared with CON are likely attributed to the use of purified cellulose in CON.

Together, these data suggest that ancient grains in a nutritionally complete diet can elicit beneficial shifts in fecal microbiota and metabolites in adult healthy dogs when compared with a rice-based diet. These beneficial shifts will likely aid in the maintenance of gut and host health. However, it is important to note that some of the observed differences between the present study and previous literature reports may be due to the differences in diet composition; health history, breed, gender of dogs; and the specific DNA primers, amplification, and sequencing methods (Simpson et al., 2002; Deng and Swanson, 2015).

### Glycemic and insulinemic responses

Baseline comparisons of postprandial responses determined that no covariate analysis was necessary and confirmed that there was no bias in starting points for any treatment groups. Regardless, results showed dietary treatment had no effect on any analyzed postprandial glucose response. The absence of a response may likely be due to the use of healthy dogs in this study. Future studies should evaluate the postprandial effects of these selected ancient grains in populations of obese or diabetic dogs, which may display more pronounced responses to these dietary treatments.

It is important to note the numerically lower values for all analyzed glucose and insulin parameters in dogs fed OG. This has been previously reported and responses are more pronounced in the literature (Braaten et al., 1991; Wood et al., 1994; Hallfrisch et al., 1995; Mäkeläinen et al., 2007; Regand et al., 2011). Those findings suggest that OG may lower the postprandial glucose response in dogs with a compromised health status. Multiple studies have also reported reductions in glycemic and insulinemic responses in diabetic and/or obese humans (Jenkins et al., 1986; Pereira et al., 2002; Panahi et al., 2007). The moderate glycemic index (RGR < 1.0) for OG suggests that it is appropriate to use in diet formulation requiring moderate glucose and energy levels (e.g., weight control and senior). The high glycemic indexes (RGR > 1.0) of AM, QU, and WPM suggest that these ancient grains are appropriate for diet formulations requiring higher glucose and energy levels (e.g., reproduction, growth, and performance). To evaluate if these patterns could be explained by diet processing parameters, glycemic response data were compared with starch cook data, and no correlations were found for any categories (data not shown). Thus, it can be inferred that the slight difference in the level of starch cook between dietary treatments was not a confounding factor nor did it have any measurable effect on the observed postprandial response of glucose or insulin.

## Conclusions

When utilized as the main carbohydrate source at relatively high inclusion levels (up to 40%) in extruded adult dog foods, ancient grains are well accepted and appropriate for adult dogs with no detrimental effects on stool quality or macronutrient digestibility. The dietary inclusion of AM and OG is specifically beneficial in shifting fermentative end products indicative of a butyrogenic effect. Although OG did not significantly impact the postprandial glycemic or insulinemic responses in healthy dogs in this study, it can be implied that this ancient grain may benefit obese, insulin resistant, and/or diabetic dogs. This benefit should be evaluated in future studies.

## Abbreviations

AHF: acid hydrolyzed fat
ATTD: apparent total tract digestibility
AUC: area under the curve
BCFA: branched-chain fatty acids
BCS: body condition score
CP: crude protein
DE: digestible energy
DM: dry matter
DMB: dry matter basis
GE: gross energy
IAUC: incremental area under the curve
ME: metabolizable energy
OM: organic matter
OTU: operational taxonomic unit
PCoA: principal coordinate analysis
PD: phylogenetic diversity
RGR: relative glycemic ratio
RIR: relative insulin ratio
SCFA: short-chain fatty acids
TDF: total dietary fiber
UniFrac: unique fraction metric
